# Structural basis of H-NS-mediated temperature-dependent stimulation of initial growth in *Escherichia coli*

**DOI:** 10.1371/journal.pone.0346655

**Published:** 2026-10-28

**Authors:** Erika Yamauchi, Hayato Fujita, Yukari Miyake, Kaneyoshi Yamamoto

**Affiliations:** 1 Department of Frontier Bioscience, Hosei University, Tokyo, Japan; 2 Research Institute of Micro-Nano Technology, Hosei University, Tokyo, Japan; Berhampur University, INDIA

## Abstract

Bacteria rapidly initiate growth when entering warm-blooded hosts to ensure reproductive success. We investigated the temperature-dependent stimulation of the specific growth rate at the onset of the logarithmic phase (defined as μ at *OD*_*ratio*_ = 0.2) in *Escherichia coli.* High-frequency automated monitoring revealed that this stimulation peaks near 37°C and is strain-specific. Comparative analysis between MG1655 and W3110 type A identified the Rac prophage as a primary genomic determinant for this acceleration. However, growth stimulation is uniquely dependent on H-NS, which we redefine as a nucleoid structural organizer. Genetic complementation and comparative analysis of nucleoid-associated proteins (NAPs), and single-cell nucleoid imaging confirmed that H-NS provides a mandatory physical scaffold specifically at host temperatures. We propose that H-NS undergoes a conformational switch to a parallel dimer form around 37°C, enabling the nucleoid to adopt the highly condensed state required for managing replication-transcription conflicts during rapid growth bursts. Notably, while MG1655 relies primarily on H-NS for this stimulation, W3110 type A requires a distributed network of NAPs, including HU and StpA, to maintain its growth velocity. This effect is environment-specific, becoming particularly significant under minimal media conditions where metabolic and structural coordination requirements are more stringent, but less pronounced in nutrient-rich conditions. These findings resolve the silencing paradox: *hns*-deficient mutants grow slowly at 37°C because they lack the structural infrastructure required to physically coordinate the rapid onset of growth, regardless of the de-repression of growth-promoting genes. We conclude that H-NS-mediated physical organization of the genome is a fundamental prerequisite for temperature-dependent growth adaptation and successful host colonization.

## Introduction

Bacterial survival depends on the ability to perceive external stimuli and translate them into biological responses. One of the most critical adaptations for *E. coli* is the ability to sense and respond to the temperature of the host gut, which typically resides around 37°C [1]. Success in these environments is often determined by the speed at which a population can initiate growth and expand its clonal presence upon entry. This rapid growth requires a highly organized and condensed genome structure to manage the high DNA concentrations and coordinate replication and transcription.

Prophages play a significant role in this adaptive landscape. Approximately 20% of the *E. coli* K-12 genome is composed of prophage DNA, much of which was historically considered junk [2,3]. However, contemporary research suggests that these cryptic prophages provide selective advantages, allowing hosts to thrive under various limiting conditions [4]. Specifically, prophages appear to stimulate host growth rates, creating a symbiotic relationship that ensures the reproductive success of both the host and the integrated viral DNA.

The modulation of these exogenous genes is primarily governed by H-NS (histone-like nucleoid-associated protein). Previous studies have established that H-NS is essential for this orderly folding of the chromosome [5]. Specifically, cells lacking H-NS are incapable of adopting the distinct, condensed nucleoid structures characteristic of rapid growth and instead retain a slow-growth type of chromosome organization even under nutrient-rich conditions [5]. While H-NS is traditionally characterized as a silencer of horizontally transferred DNA [6–9], its role as a prerequisite structural infrastructure for rapid growth is more fundamental. Previous evidence also suggests it acts as a nucleoid structural organizer required for maintaining a condensed nucleoid structure and facilitating rapid growth [10–13]. The transition from a repressive silencer to an active structural organizer is believed to be temperature-dependent. The objective of this study is to investigate how H-NS mediates the temperature-dependent stimulation of specific growth rate at the onset of the logarithmic phase through structural reorganization. We explore the hypothesis that H-NS-mediated conformational changes, specifically in the presence of prophagic elements like the Rac prophage, provide the physical framework necessary for *E. coli* to adapt to the constant temperatures of warm-blooded hosts.

## Materials and methods

### Bacterial strains and genome sequencing

The bacterial strains used in this study include *E. coli* K-12 MG1655, W3110 type A, and their derivatives (Table 1). W3110 type A was specifically selected as a comparative standard because it carries a complete set of sigma factors and a functional *rpoS* allele, unlike other W3110 laboratory stocks [14]. Genomic DNA for W3110 type A was extracted using the Wizard Genomic DNA Purification Kit (Promega, Madison, WI, USA) and whole-genome sequencing was performed via the DNBSEQ-G400 platform (MGI Tech Co., Ltd., Shenzhen, China) to map genomic differences relative to MG1655 [2,3]. The genome of the strain W3110 type A (DRA/DRA009990; RUN/DRR316917) has been published in the DDBJ Sequence Read Archive (https://ddbj.nig.ac.jp/DRASearch/).

**Table 1:** *E. coli* strains, plasmids, and oligonucleotides used in this study.

Name	Characterization	Reference
**Strains**		
MG1655	Parent strain	2
W3110 type A	Wild type, complete σ set	13
DH5α	F^-^ λ^-^ Φ80*lacZΔ*M15 Δ(*lacZYA-argF*)*U169 deoR recA1 endA1 hsdR17*(r_K_-, m_K_+) *phoA supE44 thi-1 gyrA96 relA1*	Takara Bio
MGΔhns	MG1655, *hns* (R15S, A16L, Q17X)	This study
MGΔstpA	MG1655, *stpA* (L14N, R15X)	This study
MGΔhupA	MG1655, *hupA* (Q20K, A21X)	This study
MGΔhupB	MG1655, *hupB* (K9N, I10N, A11X)	This study
MGΔfis	MG1655, *fis* (S18F, Q19N, D20Y, V21X)	This study
MGΔdps	MG1655, *dps* (introduction of TTA at 37^th^ codon)	This study
WAΔhns	W3110 typeA, *hns* (R15S, A16L, Q17X)	This study
WAΔstpA	W3110 typeA, *stpA* (L14N, R15X)	This study
WAΔhupA	W3110 typeA, *hupA* (Q20K, A21X)	This study
WAΔhupB	W3110 typeA, *hupB* (K9N, I10N, A11X)	This study
WAΔfis	W3110 typeA, *fis* (S18F, Q19N, D20Y, V21X)	This study
WAΔdps	W3110 typeA, *dps* (introduction of TTA at 37^th^ codon)	This study
**Plasmids**		
psgRNA	pMB1 *bla* P-sgRNA (sgRNA cloning vector)	14
psgRNA-hns-1	pMB1 *bla* P-sgRNA-*hns*	This study
psgRNA-stpA-2	pMB1 *bla* P-sgRNA-*stpA*	This study
psgRNA-hupA-3	pMB1 *bla* P-sgRNA-*hupA*	This study
psgRNA-hupB-1	pMB1 *bla* P-sgRNA-*hupB*	This study
psgRNA-fis-2	pMB1 *bla* P-sgRNA-*fis*	This study
psgRNA-dps-4	pMB1 *bla* P-sgRNA-*dps*	This study
pCas	*repA^ts^ kan* P_cas_-*cas9* P_araB_-*red lacI*^q^ P_trc_-sgRNA-pMB1	17
pQE80L	Expression vector	Qiagen
pQE80hns	pQE80L, *hns*	12
**Oligonucleotides**	**Sequence (5’ to 3’)**	
hns_sgRNA_N20_1	CCGCAGCGGTTTCAGCATCCGTACTCTTCGTGCGCGTTTTAGAGCTAGAA	This study
hns_sgRNA_com_1	TTCTAGCTCTAAAACGCGCACGAAGAGTACGGATGCTGAAACCGCTGCGG	This study
stpA_sgRNA_N20_2	CCGCAGCGGTTTCAGTTCGCGAGCCATCGCACGGAGTTTTAGAGCTAGAA	This study
stpA_sgRNA_com_2	TTCTAGCTCTAAAACTCCGTGCGATGGCTCGCGAACTGAAACCGCTGCGG	This study
hupA_sgRNA_N20_3	CCGCAGCGGTTTCAGCTCCAGAGCAGCTTTAGCCTGTTTTAGAGCTAGAA	This study
hupA_sgRNA_com_3	TTCTAGCTCTAAAACAGGCTAAAGCTGCTCTGGAGCTGAAACCGCTGCGG	This study
hupB_sgRNA_N20_1	CCGCAGCGGTTTCAGTTGATCGACAAGATTGCTGCGTTTTAGAGCTAGAA	This study
hupB_sgRNA_com_1	TTCTAGCTCTAAAACGCAGCAATCTTGTCGATCAACTGAAACCGCTGCGG	This study
fis_sgRNA_N20_2	CCGCAGCGGTTTCAGCGTTTCTACCGTTAACTCTCGTTTTAGAGCTAGAA	This study
fis_sgRNA_com_2	TTCTAGCTCTAAAACGAGAGTTAACGGTAGAAACGCTGAAACCGCTGCGG	This study
dps_sgRNA_N20_4	CCGCAGCGGTTTCAGAGTAGAGTTGCTGAATCGCCGTTTTAGAGCTAGAA	This study
dps_sgRNA_com_4	TTCTAGCTCTAAAACGGCGATTCAGCAACTCTACTCTGAAACCGCTGCGG	This study
hns_stop	ATGAGCGAAGCACTTAAAATTCTGAACAACATCCGTACTCTAAGTAAGTAAGCAAGAGAATGTACACTTGAAACGCTGGAAGAAATGCTGG	This study
hns_stop_com	CCAGCATTTCTTCCAGCGTTTCAAGTGTACATTCTCTTGCTTACTTACTTAGAGTACGGATGTTGTTCAGAATTTTAAGTGCTTCGCTCAT	This study
stpA_stop	TTATGTCCGTAATGTTACAAAGTTTAAATAACATTCGCACTAACTAAGTAATGGCTCGCGAATTCTCCATTGACGTTCTTGAAGAAATGCT	This study
stpA_stop_com	AGCATTTCTTCAAGAACGTCAATGGAGAATTCGCGAGCCATTACTTAGTTAGTGCGAATGTTATTTAAACTTTGTAACATTACGGACATAA	This study
hupA_stop	TGATTGATGTAATTGCAGAGAAAGCAGAACTGTCCAAAACTAAGTAAATAACTGCTCTGGAGTCCACTCTGGCTGCAATTACTGAGTCTCT	This study
hupA_stop_com	AGAGACTCAGTAATTGCAGCCAGAGTGGACTCCAGAGCAGTTATTTACTTAGTTTTGGACAGTTCTGCTTTCTCTGCAATTACATCAATCA	This study
hupB_stop	AGAGGAAGAGAAGAGTGAATAAATCTCAATTGATCGACAATAATTAAGTAAGGGCTGATATCTCTAAAGCTGCGGCTGGCCGTGCGTTAGA	This study
hupB_stop_com	TCTAACGCACGGCCAGCCGCAGCTTTAGAGATATCAGCCCTTACTTAATTATTGTCGATCAATTGAGATTTATTCACTCTTCTCTTCCTCT	This study
fis_stop	CGCGTAAATTCTGACGTACTGACCGTTTCTACCGTTAACTTAAATAATTAAGTAACCCAAAAACCCCTGCGTGACTCGGTTAAACAGGCAC	This study
fis_stop_com	GTGCCTGTTTAACCGAGTCACGCAGGGGTTTTTGGGTTACTTAATTATTTAAGTTAACGGTAGAAACGGTCAGTACGTCAGAATTTACGCG	This study
dps_stop	CAGCGAGAAAAAAGCAACAGTAGAGTTGCTGAATCGCCAGTAAGTAATTAATATCCAGTTTATTGATCTTTCTTTGATTACCAAACAAGCG	This study
dps_stop_com	CGCTTGTTTGGTAATCAAAGAAAGATCAATAAACTGGATATTAATTACTTACTGGCGATTCAGCAACTCTACTGTTGCTTTTTTCTCGCTG	This study
hns-Lux-F	TCGTCTTCACCTCGAGGTAAATGACTGCTGGTAACTATTC	This study
hns-middle	TTATTGCTTGATCAGGAAATCGTCG	This study
stpA-Lux-F	TCGTCTTCACCTCGATCGCATAGCGTATATCGCTGGCTAT	This study
stpA-middle	GCTGGCGTTTTTTACCAGCGCGTGG	This study
hupA-Lux-F	TCGTCTTCACCTCGATCCGGCAATCGATGCCTGCGTGGCG	This study
hupA-middle	GAAGCAGAGCGCCAGCCAGCATCAA	This study
hupB-Lux-F	TCGTCTTCACCTCGACGCCGAAAGATGGTCCGAGTGCCGG	This study
hupB-middle	AAGGCACATCAGTAGATGCGCCCTT	This study
fis-F	GGCTCTGACCATTCATGGCCGTACA	This study
fis-R	TTCTCATGGTCACTCCCTTTGTGAC	This study
dps-Lux-F	TCGTCTTCACCTCGAGCCGTTTCCGGGATGATTTTCCTCG	This study
dps-middle-primer	CAGCGGGTAACTTTTCAGCGGGGTT	This study

*Underline represents the sequence of a stop codon cassette.

*The *E. coli* strains and plasmids in this study could be provided from the National BioResource Project (NBRP) *E. coli* of Japan.

### Growth measurement and mathematical definitions

Bacterial growth was monitored using a TVS062CA Compact Rocking Incubator (Advantec Toyo Kaisha, Ltd., Tokyo, Japan) in M9 glucose (M9-Glc) medium at temperatures ranging from 27°C to 45°C. OD_600_ readings were recorded at 15-minute intervals (Fig 1).

**Fig 1:**
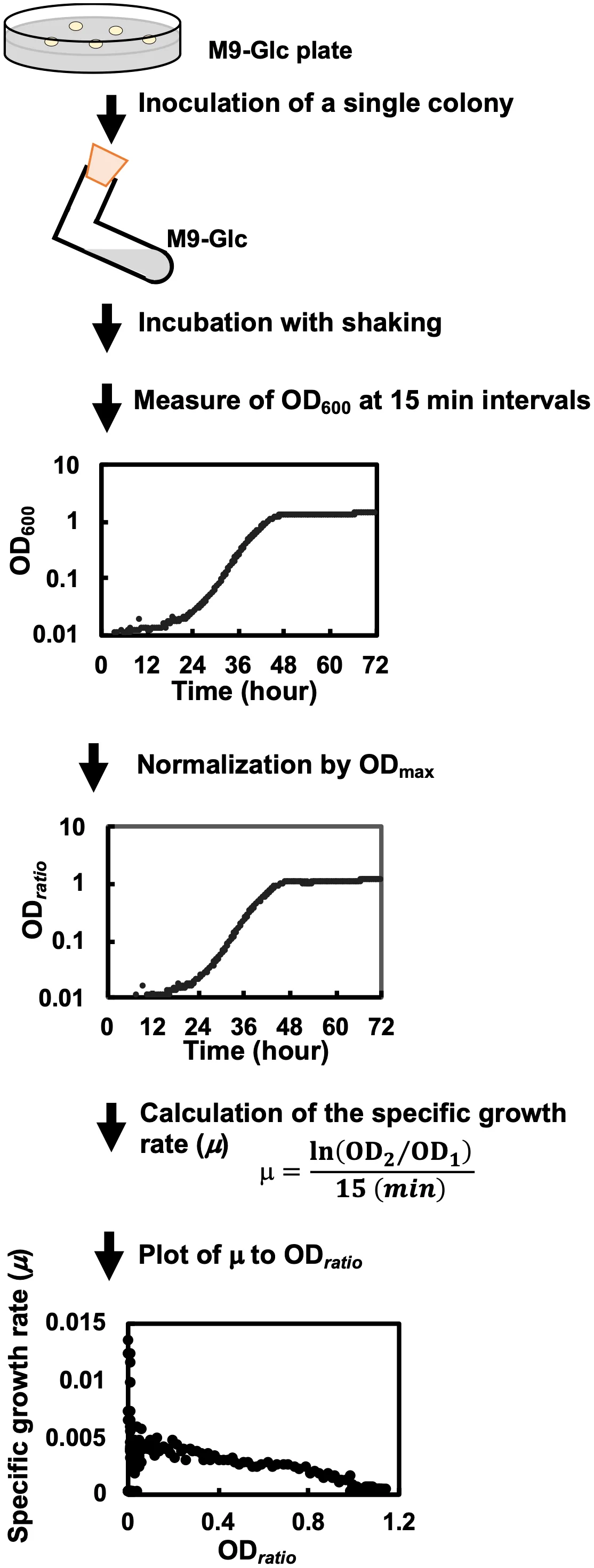
Experimental strategy for evaluating *E. coli* growth kinetics. *E. coli* growth was analyzed by quantifying the relationship between the specific growth rate and culture turbidity. A single colony was inoculated into M9-Glc medium in an L-shaped glass tube and incubated at 37°C with continuous shaking. Growth was monitored via high-frequency OD600 measurements at 15-minute intervals for up to 72 hours using a TVS062CA Compact Rocking Incubator (Advantec, Japan). To standardize growth profiles, OD600 values were normalized to the maximum observed turbidity (ODmax) to define the *ODratio* (*ODmeasured/ODmax*). The specific growth rate (μ) was calculated for each 15-minute interval using the formula μ = ln(*OD2/OD1*)/15, and subsequently plotted against the *ODratio*. Data represent findings from 12 independent colonies per strain.

- **Specific Growth Rate (μ)**: Calculated as *μ = ln(OD2/OD1)/15 min*
- **Normalization**: OD values were normalized to the maximum observed turbidity as *ODratio* = *ODmeasured* / *ODmax*
- **Specific growth rate at the onset of the logarithmic phase**: Defined as the specific growth rate at the onset of the logarithmic phase. This was calculated as the value of μ when *ODratio* = 0.2, using a logarithmic fit of the growth curve as *y = aln(x) + b*

### HoSeI method for mutant construction

Mutants for *hns, stpA, hupA, hupB, fis*, and *dps* were created using the Homologous Sequence Integration (HoSeI) method (S1 Fig) [15]. This technique utilizes CRISPR-Cas9 to induce double-strand breaks at target loci, which are then repaired by integrating a stop codon cassette containing the sequence TAAnTAAnTAA, which terminates all three reading frames. Transformants were selected via antibiotic sensitivity and confirmed through Sanger sequencing (S2 Fig).

### Microscopic observations and image analysis

Microscopic observations were performed according to the methods previously described [16] with minor modifications. Bacterial cells were grown in M9-Glc medium at 37°C to early exponential phase (OD_600_ ≈ 0.3–0.5). For nucleoid visualization, 2 mL of the culture was harvested by centrifugation (10,000 rpm, 5 min), and the cell pellet was resuspended in M9-Glc medium containing DAPI (final concentration 2 μg/mL). Following a 5-minute incubation in the dark, the cells were placed onto a glass slide, covered with a glass coverslip, and sealed with nail polish. Observations were conducted using an IX81 microscope (Olympus, Tokyo, Japan) equipped with a 100/NA 1.4 objective lens (M Plan Apochromat MPLAPON-Oil, Olympus, Tokyo, Japan) and a Retiga EXi Fast1394 CCD camera (Q Imaging, Surrey, BC, Canada). DAPI-stained cells were imaged using a U-MWU2 filter set. To determine the nucleoid occupancy ratio, the nucleoid area and total cell area were quantified using ImageJ software (National Institutes of Health, Bethesda, MD, USA) as shown in S8 Fig.

## Results

### Temperature-dependent growth patterns

The MG1655 strain exhibited a clear temperature-dependent growth response between 30°C and 42°C (S3 and S4 Figs) (Fig 2). We observed a maximum specific growth rate at the onset of the logarithmic phase followed by a temperature-dependent rate of deceleration, a phenomenon termed growth compression. At the physiological temperature of 37°C, the specific growth rate and *ODratio* followed the logarithmic fit *y = −0.005ln(x)+0.0026* (Fig 2). As the temperature approached 37°C, both the initial velocity and the rate of growth compression increased significantly, indicating a specialized adaptation to host-gut temperatures.

**Fig 2:**
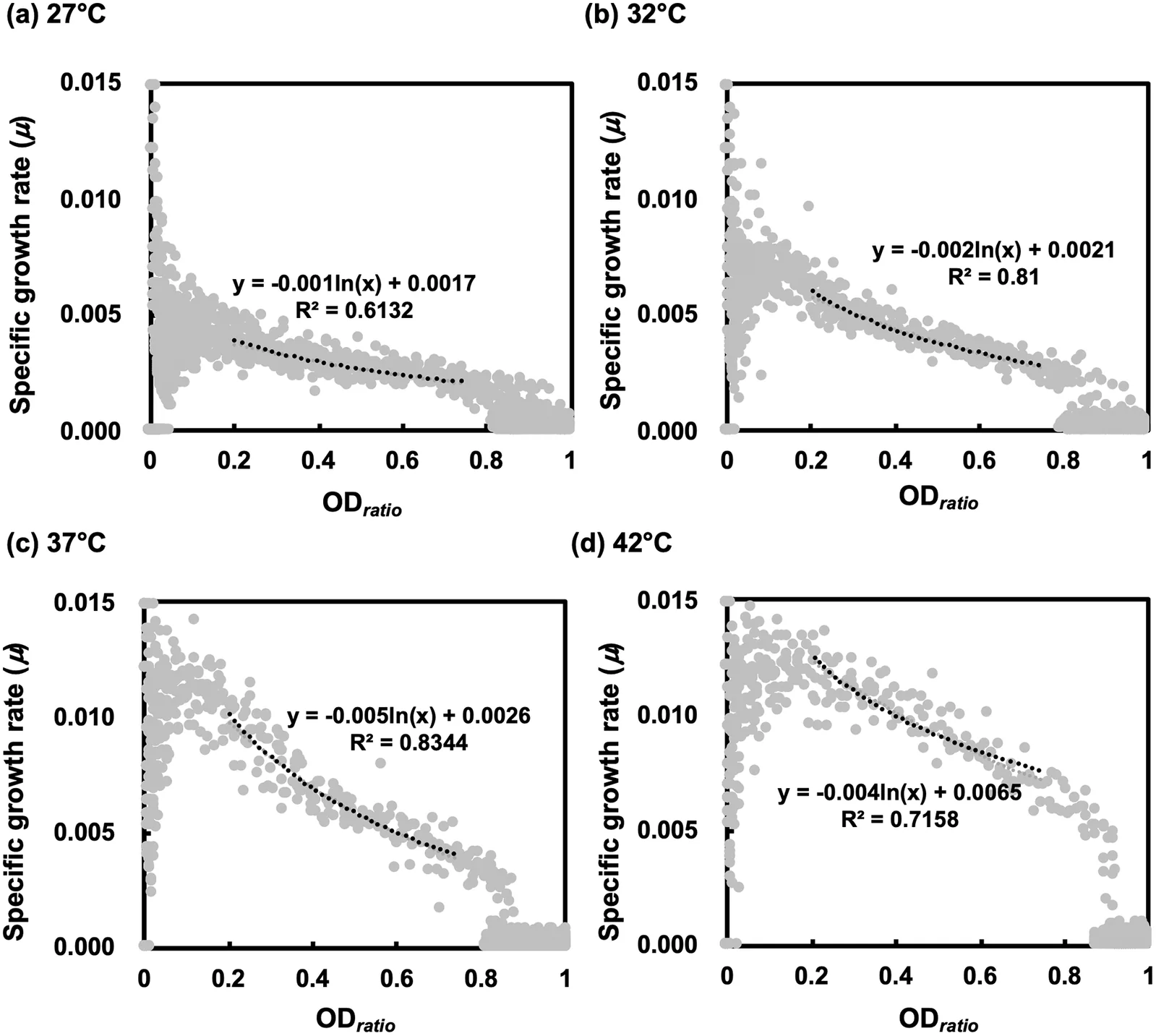
Quantitative analysis of temperature-dependent growth kinetics and the stimulation effect. The relationship between the instantaneous specific growth rate (μ) and normalized culture turbidity (*ODratio*) was evaluated across a range of temperatures. (a–d) Representative plots of μ versus *ODratio* for the parent strain MG1655 at 27°C (a), 32°C (b), 37°C (c), and 42°C (d). Individual data points (gray circles) represent measurements taken at 15-minute intervals from 12 independent cultures. The dotted lines indicate the logarithmic regression fit (y = aln(x)+b) calculated within the *ODratio* range of 0.2 to 1.0, which corresponds to the logarithmic growth phase. The regression formula and the coefficient of determination (R^2^) are displayed for each temperature condition. The specific growth rate at the onset of the logarithmic phase is statistically defined as the value of μ at *ODratio* = 0.2.

### Comparative analysis: MG1655 vs. W3110 type A

A comparison between MG1655 and W3110 type A revealed distinct kinetic profiles. Despite W3110 type A possessing a functional *rpoS* allele and a complete sigma factor set [14], it failed to match the stimulated specific growth rate at the onset of the logarithmic phase observed in MG1655 at temperatures ≥37°C (Fig 3a). While MG1655 showed marked stimulation at 37°C compared to 27°C, W3110 type A displayed nearly identical velocities at both temperatures (Fig 3a). This suggests that the presence of the *rpoS* allele is insufficient to compensate for the lack of specific growth-promoting genomic elements in the W3110 lineage.

**Fig 3:**
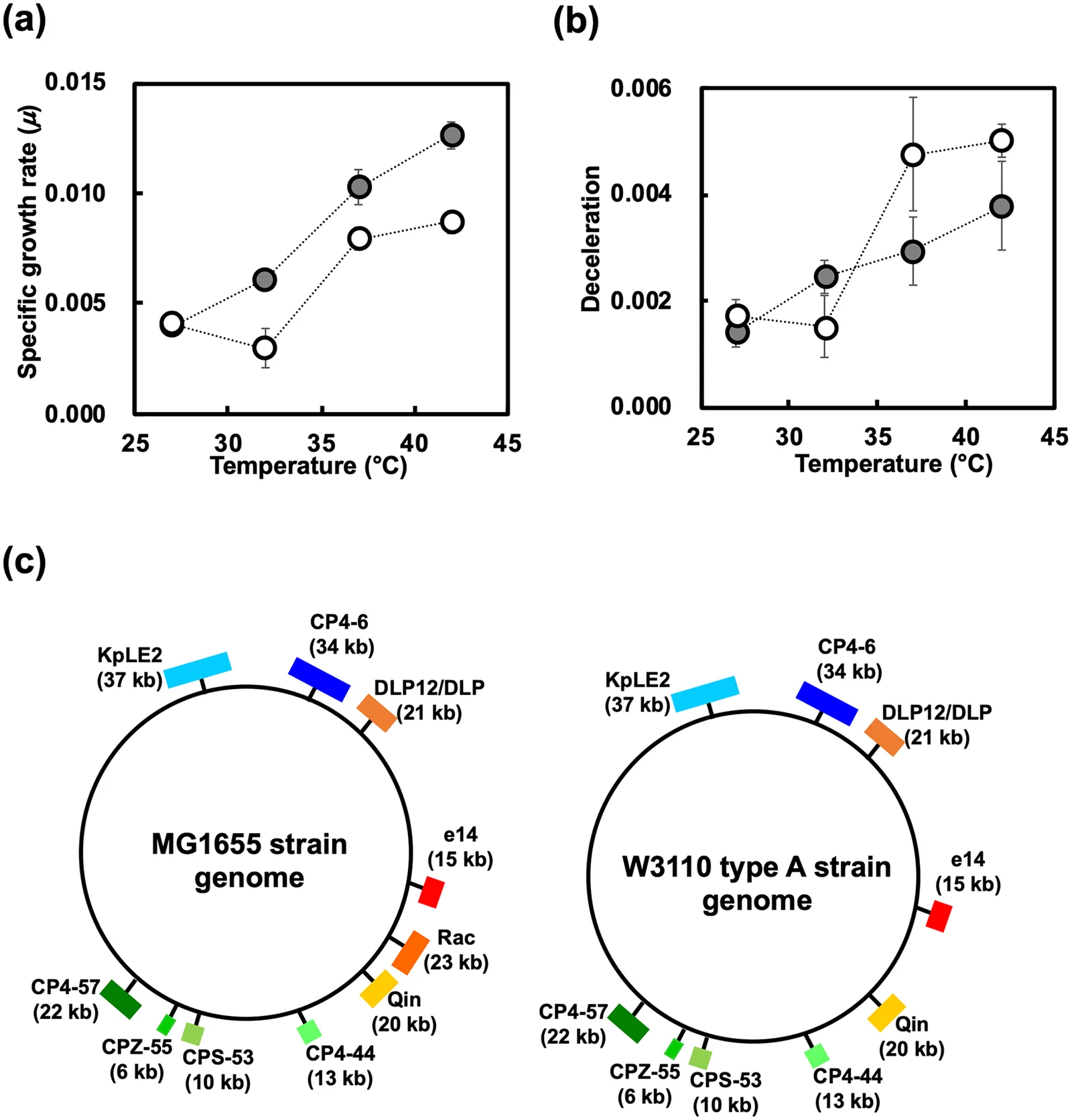
Impact of the Rac prophage on temperature-dependent growth stimulation and comparative genomic mapping. (a) Temperature-dependent profiles of the specific growth rate at the onset of the logarithmic phase (μ at *ODratio* = 0.2) for *E. coli* MG1655 (filled circles) and W3110 type A (open circles). (b) Comparison of growth deceleration rates across temperatures for MG1655 and W3110 type A. In both (a) and (b), each data point represents the mean value derived from 12 independent colonies, with error bars indicating the standard deviation. (c) Comparative circular genome maps of MG1655 (left) and W3110 type A (right). Colored boxes indicate the positions and sizes (kb) of various prophage elements. The 23-kb Rac prophage (orange box) is present in the MG1655 genome but notably absent in W3110 type A, correlating with the lack of significant growth stimulation at 37°C in the latter strain.

### Genomic mapping of the Rac prophage

Whole-genome sequencing of W3110 type A identified a major 23-kb deletion corresponding to the Rac prophage (Fig 3c). Additionally, a 6-kb region spanning *ynaJ* to *abgA* was found to be absent in W3110 type A. However, it is highly unlikely that this deletion contributes to the observed growth defect. Comprehensive growth analyses of the Keio Collection [17]—a set of in-frame single-gene knockout mutants—have previously established that the nine genes within this 6-kb region (*ynaJ, uspE, fnr, ogt, abgT, abgB, abgA, tnaB*, and *dcuA*) are non-essential and their individual deletions do not significantly impact growth rates. Since these genes do not exhibit growth-promoting properties, their absence in W3110 type A cannot explain the specific reduction in the stimulated specific growth rate at 37°C. Given the differential growth kinetics, these data indicate that the Rac prophage is a primary factor in the stimulation of specific growth rate at the onset of the logarithmic phase at host temperatures.

### H-NS essentiality and NAP comparison

To isolate the role of H-NS, we analyzed the specific growth rates at the onset of the logarithmic phase (defined as μ at *OD*_*ratio*_ = 0.2) of various nucleoid-associated protein (NAP) mutants. Both MGΔhns and WAΔhns showed the absence of detectable H-NS (S5 Fig) and exhibited significantly specific growth rates compared to their parent strains (Fig 4a). A genetic complementation assay at 37°C revealed that the growth stimulation in MGΔhns was successfully restored by the introduction of an *hns*-expression plasmid (pQE80hns), whereas the growth defect in WAΔhns was not complemented (Fig 4a, rightmost panel). Next, we evaluated whether the observed growth stimulation is dependent on nutrient availability or specific carbon sources by monitoring kinetics in M9-glycerol (M9-Gly; S6 Fig) and M9-casamino acids (M9-Cas; S7 Fig) at 37°C. Unlike the distinct profiles observed in M9-Glc medium, MG1655 and W3110 type A exhibited nearly identical specific growth rates at the onset of the logarithmic phase and comparable growth deceleration patterns in M9-Gly (S6B Fig). In M9-Cas medium, while MG1655 maintained a higher growth rate, the deletion of *hns* significantly reduced this velocity to levels comparable to the mutant in M9-Glc (S7B Fig). Notably, W3110 type A did not exhibit prominent growth stimulation even in nutrient-enriched M9-Cas, reinforcing that this strain inherently lacks the temperature-dependent acceleration observed in MG1655 (S7 Fig). These findings suggest that the growth-promoting effect mediated by H-NS and the Rac prophage is highly environment-specific. The requirement for H-NS as a nucleoid structural organizer becomes particularly critical under minimal media conditions, such as M9-Glc, where the biophysical coordination of transcription and replication is a prerequisite for overcoming metabolic constraints.

**Fig 4:**
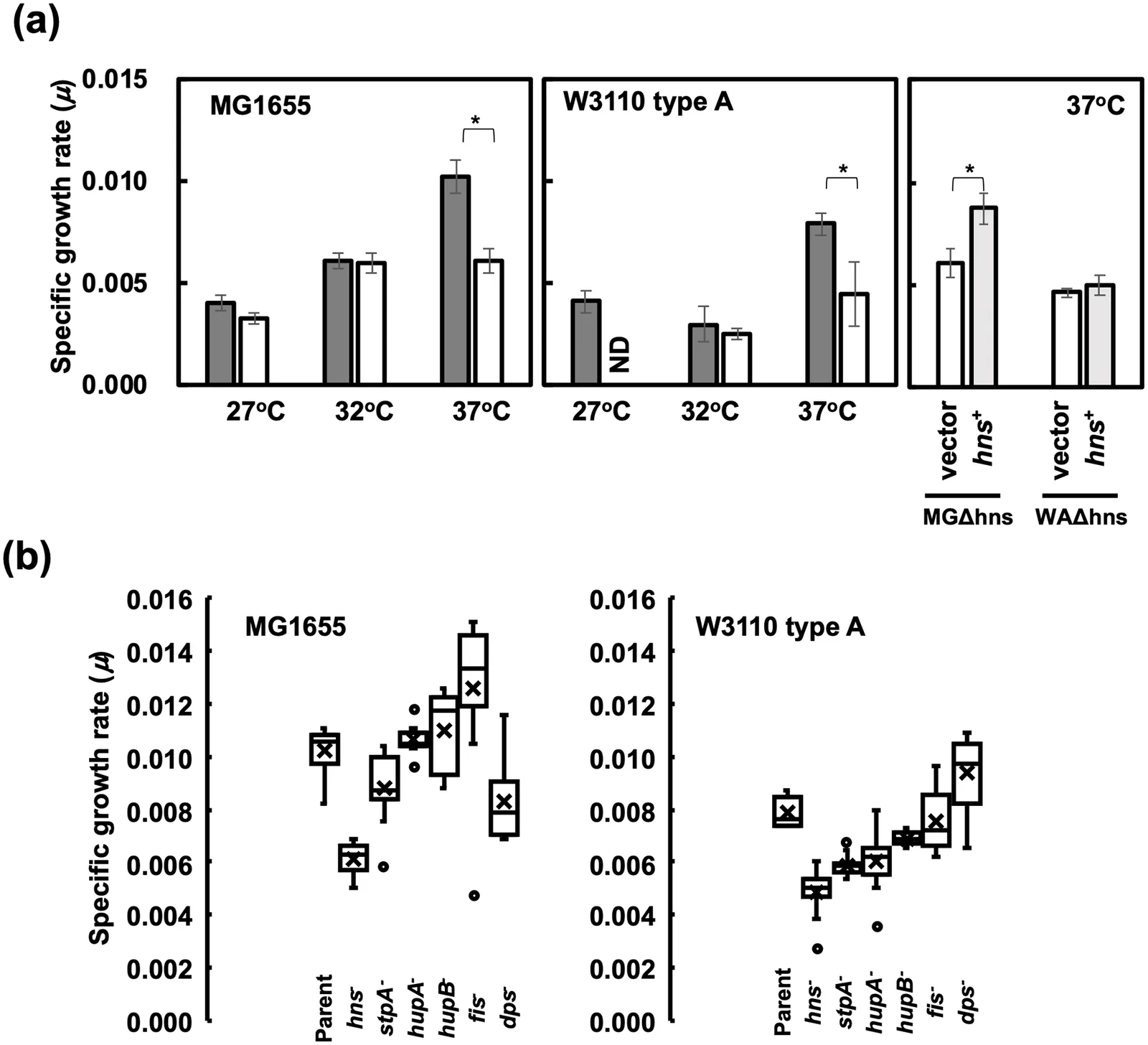
Essential role of H-NS in temperature-dependent growth stimulation and comparison with other nucleoid-associated proteins (NAPs). (a) Comparison of the specific growth rate at the onset of the logarithmic phase (defined as μ at *ODratio* = 0.2) among parent strains (MG1655 and W3110 type A; gray bars) and their respective *hns*-deficient mutants (MGΔhns and WAΔhns; open bars) at 27°C, 32°C, and 37°C. MGΔhns and WAΔhns haboring either the empty vector (pQE80L; open bars) or the *hns*-expression plasmid (pQE80hns; light gray bars) [12] were compared. (b) Specific growth rates of various NAP mutants (*hns*^*-*^*, stpA*^*-*^*, hupA*^*-*^*, hupB*^*-*^*, fis*^*-*^, and *dps*^*-*^) at 37°C. These results demonstrate that while several NAPs contribute to nucleoid architecture, H-NS is uniquely essential as a nucleoid structural organizer for stimulating initial growth in response to host-gut temperatures. In panel (a), bars represent the mean of 12 independent colonies for the two graphs on the right and 3 independent colonies for the graph on the left, with error bars indicating the standard deviation. In panel (b), box plots represent the distribution from 12 independent measurements. Asterisks indicate *p* < 0.05 based on Student’s t-test. ND: not determined.

The dependency on other NAPs for growth stimulation differed between the two parent lineages (Fig 4b). In the MG1655 background, mutants of *hupA, hupB, fis,* and *dps* did not show a similar essentiality for growth acceleration; specifically, *fis* mutants maintained or even exceeded the parent strain’s specific growth rate, and *dps* mutants showed only marginal defects. In contrast, in the W3110 type A background, growth velocities were significantly reduced not only in *hns* mutants but also in *stpA, hupA,* and *hupB* mutants, while *fis* and *dps* deletions had little effect. These findings indicate that while H-NS is the primary determinant for growth at 37°C in MG1655, W3110 type A requires additional NAPs—specifically the H-NS homolog StpA and the HU protein (the HupA-HupB heterodimer)—to maintain its growth rate. Nevertheless, across all tested conditions and backgrounds, the defect in *hns* mutants remained the most severe. This confirms that the *hns* gene is uniquely essential as a nucleoid structural organizer for the temperature-dependent stimulation of the specific growth rate at the onset of the logarithmic phase.

### Microscopic observation of nucleoid organization

To address whether the reduced growth velocity in mutants correlates with observable structural defects, we performed single-cell nucleoid imaging using DAPI staining in M9-Glc medium at 37°C (S8 Fig). Quantitative analysis of the nucleoid-to-cell area ratio revealed no significant difference between MG1655 and MGΔhns (*p* = 0.61), although MG exhibited a slight tendency toward higher occupancy. In the W3110 background, a marginal but statistically significant difference was observed (*p* = 0.043), with WAΔhns showing a lower occupancy ratio compared to the parent strain. These results in minimal medium are consistent with previous findings by Helgesen et al. (2016), who reported that H-NS-dependent nucleoid condensation is nutrient-dependent and less pronounced under poor-nutrient conditions [5]. The fact that growth stimulation is severely impaired even when macroscopic nucleoid occupancy remains relatively stable suggests that the H-NS-mediated physical scaffold is required for fine-scale coordination of transcription and replication long before large-scale genomic condensation becomes visible.

## Discussion

### The structural organizer model

The finding that *hns* deletion resulted in the most severe growth defects in both MG1655 and W3110 type A lineages (Fig 4) reinforces the universality of the H-NS structural organizer model. H-NS exists in two homodimeric states: an anti-parallel form that induces higher-order multimerization and gene silencing, and a parallel form that promotes unfolding [10–12]. At approximately 37°C, H-NS transitions toward the parallel form, acting as a nucleoid structural organizer [13]. Biochemically, this switch facilitates growth by providing a physical scaffold that coordinates the spatial organization of the nucleoid, which is essential for managing replication-transcription conflicts during the rapid burst of cellular activity [5,13]. While it has been suggested that the slow growth of Δ*hns* strains results from severe metabolic deregulation and fitness defects [5], our data indicate a more fundamental biophysical requirement. Although H-NS is traditionally characterized as a silencer, our data confirm its unique and indispensable role as a physical scaffold at host temperatures (~37°C), serving as a prerequisite for successful host colonization and growth adaptation.

### Biophysical requirements for growth stimulation: Insights from nucleoid imaging

To further substantiate the structural organizer model, we performed single-cell nucleoid imaging using DAPI staining under our primary experimental condition (M9-Glc at 37°C). Quantitative analysis revealed that the nucleoid-to-cell area ratio remained relatively stable, with no significant difference observed in the MG1655 background (S8 Fig). While *hns* deficiency is known to cause severe condensation defects in nutrient-rich media, our results in minimal medium are perfectly consistent with the findings of Helgesen et al. (2016), who reported that H-NS-dependent macroscopic nucleoid condensation is nutrient-dependent and becomes nearly indistinguishable between wild-type and hns mutants under poor-nutrient conditions [5]. The fact that temperature-dependent growth stimulation is severely impaired in Δ*hns* mutants even when macroscopic nucleoid occupancy remains stable suggests a critical insight: the H-NS-mediated physical scaffold is required for the fine-scale biophysical coordination of transcription and replication long before large-scale genomic condensation becomes visible. In minimal media like M9-Glc, where metabolic constraints are stringent, this invisible structural infrastructure provided by the H-NS parallel-dimer form is a prerequisite for managing the biophysical stresses of initiating growth. Thus, our microscopic data not only align with established literature but also reinforce the model that H-NS acts as a mandatory structural organizer whose role is particularly vital for overcoming the initial barriers to rapid growth adaptation.

### MG1655: A specialized strategy driven by the Rac prophage

The adaptive strategy of MG1655 appears highly specialized and centered on the Rac prophage. At host temperatures, the H-NS conformational switch to the parallel form specifically unlocks the explosive growth potential harbored within the Rac elements. In this lineage, H-NS acts as the primary structural hub for growth stimulation. Consequently, the contribution of other NAPs, such as HU or StpA, is relatively marginal, as H-NS alone provides the necessary coordination for the rapid burst of transcription and replication triggered by the prophage. The successful restoration of growth by *hns* complementation in MGΔhns further confirms that H-NS is the direct determinant of this stimulated growth state (Fig 4a).

### Distinct functional attributes: Metabolic utilities versus growth-promoting elements

A critical consideration in identifying the genomic basis of growth stimulation is the 6-kb deletion (*ynaJ*–*abgA*) also present in the W3110 type A lineage. However, we argue that the functional attributes of the genes in this region are fundamentally distinct from those of the Rac prophage. The nine genes within the 6-kb region (*ynaJ, uspE, fnr, ogt, abgT, abgB, abgA, tnaB*, and *dcuA*) primarily function as auxiliary metabolic transporters and sensors, such as the anaerobic regulator *fnr* or the nutrient transporters *tnaB* and *dcuA*. Systematic growth data from the Keio Collection confirms that none of these genes individually contribute to growth rate stimulation [17].

In contrast, prophage elements like the Rac prophage are evolutionarily recognized as growth-promoting elements that provide a selective advantage by directly stimulating the host’s reproduction. Crucially, H-NS preferentially targets horizontally acquired, AT-rich sequences—typical of prophages like Rac—as the primary sites for its structural organizing function [4]. Given that the 23-kb Rac prophage represents a massive structural target for H-NS-mediated organization at 37°C, its absence is a biologically more plausible explanation for the lost growth stimulation than the deletion of secondary metabolic utilities. Thus, the Rac prophage acts as the primary weapon whose potential is unlocked by H-NS, while the 6-kb region lacks both the functional and structural properties necessary for such an adaptive response.

### W3110 type A: A distributed defense strategy via multiple NAPs

In contrast, W3110 type A, which lacks the Rac prophage, does not exhibit the same level of temperature-dependent growth stimulation (Fig 3a). As established by Helgesen et al. (2016), rapid growth requires high-order genome condensation to manage DNA density and prevent replication-transcription conflicts [5]. Our results indicate that W3110 maintains this essential structural infrastructure through a distributed strategy. Unlike MG1655, W3110 requires a broader network of NAPs—specifically H-NS, its homolog StpA, and the HU protein (HupA-HupB heterodimer)—to maintain its growth velocity at 37°C (Fig 4b). The comparable growth defects observed in WAΔhns and WAΔhupB suggest a high degree of structural interdependence in the W3110 lineage; in the absence of prophage-mediated acceleration, the biophysical framework of W3110 appears equally sensitive to the loss of basic condensing agents like HU. The failure of *hns* to complement growth in WAΔhns (Fig 4a) further suggests that the structural requirements in the W3110 lineage involve a more intricate and interdependent interplay of multiple NAPs that cannot be restored by H-NS alone once the balance is disrupted.

### Evolutionary divergence in structural maintenance

These distinct phenotypes demonstrate how specific genomic elements, such as the presence or absence of prophages, dictate the cell’s internal structural maintenance strategy and its degree of dependency on various NAPs. This reflects an evolutionary adaptation where different *E. coli* lineages have optimized their biophysical frameworks for host colonization: MG1655 utilizes a highly specialized, H-NS-centric mechanism to exploit prophagic potential, while W3110 adopts a more robust, distributed network to ensure genomic stability under similar thermal conditions.

## Supporting information

S1 Fig Construction of *hns*-deficient mutants. The *hns*-deficient strains were constructed using the Homologous Sequence Integration (HoSeI) method, as previously described [14]. Schematic representation of the HoSeI strategy. The sgRNA expression plasmid, psgRNA_hns_1, was introduced into *E. coli* MG1655 and W3110 type A strains harboring pCas, along with a synthetic DNA fragment. This fragment was designed to repair the double-strand break induced by CRISPR-Cas9 while simultaneously integrating a stop codon cassette (TAAnTAAnTAA) to terminate all three reading frames (Table 1). Co-transformation with psgRNA_hns_1 and the repair fragment yielded transformants on LB agar containing ampicillin (100 μg/mL) at 30°C [18]. Following the established protocol, IPTG was added to the medium to induce the expression of a gRNA targeting the pMB1 replication origin, leading to the intracellular digestion of psgRNA_hns_1. Ampicillin-sensitive and kanamycin-sensitive colonies were subsequently selected at 42°C to confirm the loss of both the sgRNA and pCas plasmids, respectively.
(TIFF)

S2 Fig Verification of nucleoid-associated protein (NAP) mutant constructions. In addition to the *hns*-deficient strains (A) described in S1 Fig, the *stpA* (B), *hupA* (C), *hupB* (D), *fis* (E), and *dps* (F) genes were disrupted in *E. coli* MG1655 and W3110 type A using the HoSeI method. (a) Schematic representation of the target region for each gene disruption. The genomic sequences of the parent *E. coli* strains (b and d) and the generated mutants (c and e) were confirmed via Sanger DNA sequencing, as described in the Materials and Methods. Briefly, genomic DNA was extracted from the transformants and used as a template for PCR amplification of the target sequences (primers are listed in Table 1). Subsequent Sanger sequencing confirmed the precise integration of the stop codon cassette (TAAnTAAnTAA) at each intended locus.
(TIFF)

S3 Fig Raw growth kinetics of *E. coli* MG1655 and the *hns*-deficient mutant. Growth profiles of the parent strain *E. coli* MG1655 (A) and the *hns*-deficient mutant (B) were monitored across a range of temperatures: 27°C (a), 32°C (b), 37°C (c), 42°C (d), and 45°C (e). Culturing was performed in M9-Glc medium using a TVS062CA Compact Rocking Incubator, with high-frequency OD_600_ measurements recorded every 15 minutes for up to 72 hours. Each panel displays the OD_600_ values from 12 independent colonies (indicated by different shades of grey) alongside the continuous temperature monitoring data (blue line). Note that growth was effectively inhibited at 45°C (e) in both strains.
(TIFF)

S4 Fig Raw growth kinetics of *E. coli* W3110 type A and the *hns*-deficient mutant. Growth profiles of the parent strain *E. coli* W3110 type A (A) and the *hns*-deficient mutant (WAΔhns) (B) were monitored across a range of temperatures: 27°C (a), 32°C (b), 37°C (c), 42°C (d), and 45°C (e). Culturing was performed in M9-Glc medium using a TVS062CA Compact Rocking Incubator, with high-frequency OD_600_ measurements recorded every 15 minutes for up to 72 hours. Each panel displays the OD_600_ values from 12 independent colonies (indicated by different shades of grey) alongside the continuous temperature monitoring data (blue line). Consistent with the results for MG1655, growth was effectively inhibited at 45°C (e) in both strains.
(TIFF)

S5 Fig Western blot analysis of H-NS protein expression in parent strains and *hns*-deficient mutants. Western blotting of *Escherichia coli* H-NS was performed as previously described [12]. Briefly, cells of MG1655, MGΔhns, W3110 type A, and WAΔhns grown in LB medium at 37°C were harvested by centrifugation, resuspended in lysis buffer containing 8 M urea, and disrupted via sonication. After centrifugation, equivalent volumes of the supernatant were subjected to 18% SDS-PAGE alongside a prestained protein marker (Nacalai Tesque, Kyoto, Japan). Proteins were transferred to PVDF membranes using an iBlot semi-dry transfer apparatus (Invitrogen, Carlsbad, CA, USA). Membranes were immuno-detected with anti-H-NS rabbit serum and HRP-conjugated anti-rabbit IgG secondary antibodies (Nacalai Tesque), followed by development with a chemiluminescence kit (Nacalai Tesque). Images were captured and analyzed using a LAS-4000 IR multi-color imager (Fujifilm, Tokyo, Japan).
(TIFF)

S6 Fig Comparative growth kinetics of *E. coli* MG1655 and W3110 type A in M9 glycerol medium. [A] Growth profiles of *E. coli* parent strains and *hns*-deficient mutants: MG1655 (a), MGΔhns (b), W3110 type A (c), and WAΔhns (d). Culturing was performed at 37°C in M9 medium supplemented with 0.2% glycerol (M9-Gly) as the sole carbon source, using a TVS062CA Compact Rocking Incubator (Advantec Toyo Kaisha, Ltd., Tokyo, Japan). High-frequency OD_600_ measurements were recorded every 10 minutes for up to 48 hours. Each panel displays values from three independent colonies, indicated by different shades of grey. [B] Specific growth rate at the onset of the logarithmic phase (defined as μ at *OD*_*ratio*_ = 0.2) for MG1655, MGΔhns, W3110 type A, and WAΔhns at 37°C. Each bar represents the mean value derived from three independent colonies, with error bars indicating the standard deviation. *P* values were calculated using Student’s *t*-test.
(TIFF)

S7 Fig Comparative growth kinetics of *E. coli* MG1655 and W3110 type A in M9 casamino acid medium. [A] Growth profiles of *E. coli* parent strains and *hns*-deficient mutants: MG1655 (a), MGΔhns (b), W3110 type A (c), and WAΔhns (d). Culturing was performed at 37°C in M9 medium supplemented with 0.2% casamino acid (M9-Cas) as the sole carbon source, using a TVS062CA Compact Rocking Incubator (Advantec Toyo Kaisha, Ltd., Tokyo, Japan). High-frequency OD_600_ measurements were recorded every 10 minutes for up to 48 hours. Each panel displays values from three independent colonies, indicated by different shades of grey. [B] Specific growth rate at the onset of the logarithmic phase (defined as μ at *OD*_*ratio*_ = 0.2) for MG1655, MGΔhns, W3110 type A, and WAΔhns at 37°C. Each bar represents the mean value derived from three independent colonies, with error bars indicating the standard deviation. *P* values were calculated using Student’s *t*-test.
(TIFF)

S8 Fig Microscopic analysis of nucleoid-to-cell area ratio in parent strains and *hns*-deficient mutants. Single-cell nucleoid imaging was performed using DAPI staining for *Escherichia coli* MG1655, MGΔhns, W3110 type A, and WAΔhns grown in M9-Glc medium at 37°C. Cells were harvested at early exponential phase (0.3–0.5), resuspended in M9-Glc medium containing DAPI (final concentration 2 g/mL), and observed via fluorescence microscopy. The nucleoid area and total cell area were quantified using ImageJ to determine the nucleoid occupancy ratio. Box plots represent the distribution of ratios for MG1655 (n = 50), MGΔhns (n = 45), W3110 type A (n = 51), and WAΔhns (n = 48). Crosses (x) and horizontal lines within boxes indicate the mean and median values, respectively. *P*-values were calculated using Student’s *t*-test.
(TIFF)
