# Supplementary material for: Structural basis of H-NS-mediated temperature-dependent stimulation of initial growth in Escherichia coli
Source: PLoS One. 2026 Oct 28;21(10):e0346655. doi: 10.1371/journal.pone.0346655 (PMC13716232; doi:10.1371/journal.pone.0346655)
Supplement: Supplementary file 1 [file pone.0346655.s010.docx]

**Response to Reviewers'**

**Note on data updates:** During the revision process, we conducted a rigorous re-evaluation of our growth rate calculation scripts. We identified and corrected a previous inconsistency in the calculation of instantaneous μ values. Consequently, the regression formulas (e.g., y=−0.005ln(x)+0.0026 at 37°C) and the specific growth rate data points in Fig 2 and Fig 4 have been updated. These refined values provide a more precise representation of the kinetic differences between the strains.

**Response to Reviewer #1**

**Comment 1:** *Role of the Rac prophage versus the 6-kb deletion (ynaJ to abgA).*

**Response:** We agree that the 6-kb deletion is a relevant genomic difference. However, systematic growth data from the Keio Collection—a comprehensive set of single-gene knockout mutants—confirm that none of the nine genes in this region (*ynaJ, uspE, fnr, ogt, abgT, abgB, abgA, tnaB,* and *dcuA*) individually contribute to the stimulation of growth rate. These are primarily auxiliary metabolic genes. In contrast, the Rac prophage is a recognized growth-promoting element whose presence correlates with the unique stimulation observed in MG1655.

**Comment 2:** *Comparison of growth velocities in the W3110 background.*

**Response:** We have expanded the Discussion to explain the comparable growth defects in WA and WA. Unlike MG1655, which utilizes a specialized strategy centered on H-NS and Rac, W3110 type A relies on a distributed network of NAPs, including HU and StpA. In this lineage, the biophysical framework is more sensitive to the loss of basic condensing agents like HU (*hupB*), making the structural infrastructure interdependent.

**Comment 3:** *Need for additional experiments to support statements on page 6.*

**Response:** To provide direct experimental support, we performed genetic complementation assays at 37°C. The specific growth rate was successfully restored in MG by an *hns*-expression plasmid. This confirms H-NS's direct role in growth stimulation.

**Comment 4:** *Pleiotropic effects and evidence for H-NS as a "nucleoid structural organizer."*

**Response:** We acknowledge the pleiotropic nature of H-NS deletions. To substantiate the structural organizer model, we conducted single-cell nucleoid imaging using DAPI staining. Our results align with the model that H-NS provides a mandatory physical scaffold for coordinating transcription and replication during the rapid onset of growth.

**Minor observation 1:** *Instrument manufacturers and countries.*

**Response:** We have updated the Materials and methods to include the make and country for all instruments and chemicals (e.g., Advantec [Japan], MGI Tech [China], Olympus [Japan]).

**Minor observation 2:** *Location of Table 1.*

**Response:** Per the journal requirements, Table 1 has been incorporated directly into the main manuscript within the Materials and methods section.

**Response to Reviewer #2 (Dr. Abhinav Saurabh)**

We are deeply grateful for Dr. Saurabh’s thorough review and his "Experimental recommendations," which have been instrumental in refining our model.

**Comment 1:** *H-NS as a xenogeneic silencer.*

**Response:** We have revised the Introduction and Discussion to explicitly acknowledge H-NS's well-established role as a global silencer of foreign/AT-rich DNA.

**Comment 2:** *Metabolic deregulation and citation of Helgesen et al. (2016).*

**Response:** We have now integrated Helgesen et al. (2016) as a core reference. We discuss our results in the context of their finding that *hns* mutants adopt a "slow-growth" type of chromosome organization even in rich media.

**Comment 3:** *Failure to cite other researchers.*

**Response:** We have broadened our literature review and included additional citations concerning H-NS's structural roles and its interactions with other NAPs.

**Comment 4:** *References section size.*

**Response:** The references section has been expanded from 14 to 18 core references, including seminal works on H-NS architecture and growth kinetics.

**Comment 5:** *Highlighting the novelty.*

**Response:** We have clarified the novelty of our work in the Abstract and Discussion: defining H-NS as a temperature-dependent structural prerequisite that specifically coordinates the growth-promoting potential of prophagic elements like Rac.

**Comment 6:** *Syntax and grammatical errors.*

**Response:** The entire manuscript, including the first paragraph of the Results, has undergone professional English language editing.

**Experimental Recommendation 1:** *Genetic complementation assay.*

**Response:** We have added complementation data in Fig 4a. Growth stimulation in MGΔhns was restored by the *hns*-expression plasmid, whereas the lack of recovery in WAΔhns reinforces our distributed network model.

**Experimental Recommendation 2:** *Western blotting to confirm true null mutant.*

**Response:** We performed Western blotting using anti-H-NS rabbit serum. As shown in S5 Fig, H-NS protein is completely absent in our Δ*hns* mutants, confirming the efficacy of the HoSeI method.

**Experimental Recommendation 3:** *Microscopic validation of chromosomal defects.*

**Response:** We performed DAPI staining and ImageJ analysis to quantify the nucleoid-to-cell area ratio (S8 Fig). Our findings in minimal medium show that the growth defect occurs even before macroscopic condensation defects become indistinguishable, supporting the need for a fine-scale structural scaffold.

**Experimental Recommendation 4:** *Alternate carbon/nitrogen sources.*

**Response:** We monitored growth in M9-Glycerol (S6 Fig) and M9-Casamino acids (S7 Fig). These results demonstrate that the H-NS-mediated stimulation is environment-specific and most critical under the stringent metabolic conditions of M9-Glc.
