# Supplementary material for: Structural basis of H-NS-mediated temperature-dependent stimulation of initial growth in Escherichia coli
Source: PLoS One. 2026 Oct 28;21(10):e0346655. doi: 10.1371/journal.pone.0346655 (PMC13716232; doi:10.1371/journal.pone.0346655)
Supplement: Peer review history

# Peer review: Decision Letter 0

**DOI:** 10.1371/journal.pone.0346655.r001

**Authors:** Saswat Mohapatra

25 May 2026

Dear Dr. YAMAMOTO,

plosone@plos.org. When you're ready to submit your revision, log on to https://www.editorialmanager.com/pone/ and select the 'Submissions Needing Revision' folder to locate your manuscript file.

- A letter that responds to each point raised by the academic editor and reviewer(s). You should upload this letter as a separate file labeled 'Response to Reviewers'.
- A marked-up copy of your manuscript that highlights changes made to the original version. You should upload this as a separate file labeled 'Revised Manuscript with Track Changes'.
- An unmarked version of your revised paper without tracked changes. You should upload this as a separate file labeled 'Manuscript'.

If you would like to make changes to your financial disclosure, please include your updated statement in your cover letter. Guidelines for resubmitting your figure files are available below the reviewer comments at the end of this letter.

If applicable, we recommend that you deposit your laboratory protocols in protocols.io to enhance the reproducibility of your results. Protocols.io assigns your protocol its own identifier (DOI) so that it can be cited independently in the future. For instructions see: https://journals.plos.org/plosone/s/submission-guidelines#loc-laboratory-protocols. Additionally, PLOS ONE offers an option for publishing peer-reviewed Lab Protocol articles, which describe protocols hosted on protocols.io. Read more information on sharing protocols at https://plos.org/protocols?utm_medium=editorial-email&utm_source=authorletters&utm_campaign=protocols.

As the corresponding author, your ORCID iD is verified in the submission system and will appear in the published article. PLOS supports the use of ORCID, and we encourage all coauthors to register for an ORCID iD and use it as well. Please encourage your coauthors to verify their ORCID iD within the submission system before final acceptance, as unverified ORCID iDs will not appear in the published article. *Only* the individual author can complete the verification step; PLOS staff *cannot* verify ORCID iDs on behalf of authors.

We look forward to receiving your revised manuscript.

Kind regards,

Saswat Mohapatra, Ph.D.

Academic Editor

PLOS One

Journal Requirements:

When submitting your revision, we need you to address these additional requirements.

1. Please ensure that your manuscript meets PLOS ONE's style requirements, including those for file naming. The PLOS ONE style templates can be found at

https://journals.plos.org/plosone/s/file?id=wjVg/PLOSOne_formatting_sample_main_body.pdf and

https://journals.plos.org/plosone/s/file?id=ba62/PLOSOne_formatting_sample_title_authors_affiliations.pdf

2. We note that the grant information you provided in the ‘Funding Information’ and ‘Financial Disclosure’ sections do not match.

When you resubmit, please ensure that you provide the correct grant numbers for the awards you received for your study in the ‘Funding Information’ section.

3. Thank you for stating the following financial disclosure: [This work was supported by a Research Fellowship for Young Scientists from the Japan Society for Promotion of Science to YM [JP18J14641], a Grant-in-Aid for Scientific Research (C) [20K05795], the MEXT-Supported Program for the Strategic Research Foundation at Private Universities, the Takahashi Industrial and Economic Research Foundation, and the Japan Keirin Autorace Foundation to KY.].

Please state what role the funders took in the study.  If the funders had no role, please state: ""The funders had no role in study design, data collection and analysis, decision to publish, or preparation of the manuscript.""

If this statement is not correct you must amend it as needed.

Please include this amended Role of Funder statement in your cover letter; we will change the online submission form on your behalf.

4. When completing the data availability statement of the submission form, you indicated that you will make your data available on acceptance. We strongly recommend all authors decide on a data sharing plan before acceptance, as the process can be lengthy and hold up publication timelines. Please note that, though access restrictions are acceptable now, your entire data will need to be made freely accessible if your manuscript is accepted for publication. This policy applies to all data except where public deposition would breach compliance with the protocol approved by your research ethics board. If you are unable to adhere to our open data policy, please kindly revise your statement to explain your reasoning and we will seek the editor's input on an exemption. Please be assured that, once you have provided your new statement, the assessment of your exemption will not hold up the peer review process.

5. Please include your tables as part of your main manuscript and remove the individual files. Please note that supplementary tables (should remain/ be uploaded) as separate "supporting information" files".

6. Please include captions for your Supporting Information files at the end of your manuscript, and update any in-text citations to match accordingly. Please see our Supporting Information guidelines for more information: http://journals.plos.org/plosone/s/supporting-information.

7. If the reviewer comments include a recommendation to cite specific previously published works, please review and evaluate these publications to determine whether they are relevant and should be cited. There is no requirement to cite these works unless the editor has indicated otherwise.

Additional Editor Comments:

The authors may look into the comments of the reviewers and revise the manuscript to address the comments.

[Note: HTML markup is below. Please do not edit.]

Reviewers' comments:

Reviewer's Responses to Questions

**Comments to the Author**

1. Is the manuscript technically sound, and do the data support the conclusions?

Reviewer #1: Partly

Reviewer #2: No

**********

2. Has the statistical analysis been performed appropriately and rigorously?

Reviewer #1: Yes

Reviewer #2: Yes

**********

3. Have the authors made all data underlying the findings in their manuscript fully available?

The PLOS Data policy

Reviewer #1: Yes

Reviewer #2: Yes

**********

4. Is the manuscript presented in an intelligible fashion and written in standard English?

Reviewer #1: Yes

Reviewer #2: No

**********

Reviewer #1: The authors in the article have compared growth rate of E. coli MG1655 and E. coli W3110 type A strains to decode the role of H-NS as a nucleoid structural organizer affecting temperature dependent stimulation of growth. The selection of W3110 is based on presence of functional rpoS and a 23kb Rac prophage deletion in the chromosome. The growth analysis is detailed and statistical analysis of the data has been presented. Given these strong points, the manuscript weakness lies in lack of gene deletion and complementation studies. It is observed that the results obtained are overinterpreted without additional experiments to substantiate the conclusions as detailed below:

1. Page 5, lines 9 -10: The statement “Given the differential …….phase at host temperatures.

It is important that Rac deletion and complementation studies are undertaken to substantiate the statement. Given the fact that W3110 also lacks 6kb region spanning ynaJ to abgA, the conclusion of role of Rac alone in stimulating specific growth rate at onset of logarithmic phase is questionable.

2. Page 5, line 13-21: The growth rate of MG1655 and MGΔhns at 27ºC is not significantly different and that of W3110Δhns is not determined. Difference in growth velocities are observed only at 37 ºC. Further, initial growth velocities of W3110Δhns and W3110ΔhupB are comparable. Could the Author’s explain this observation.

3. Page 6, line 7-9: Author’s need to perform additional experiments to support the statement made.

4. Deletion mutants of H-NS exhibit pleiotropic effects. Given the aforementioned fact, strong experimental evidence is needed to substantiate it’s significant contribution as nucleoid structural organizer affecting temperature dependent stimulation of growth.

5. Discussion needs to revised by analysing the findings in context of the available literature.

Minor observations:

1. Make and Country of respective instruments/ chemical used must be included

2. Table -1 could not be located in the manuscript/supplementary data. Has it been uploaded?

Reviewer #2: The authors discuss the role of H-NS in temperature-dependent growth rate through structural reorganization.

• The authors overlook the broader role of H-NS, including its function as a xenogeneic silencer that can repress nearly 5% of the genome (foreign or energy-rich AT-rich DNA)

• In essence, knockdown of H-NS has shown that cellular growth is marked by severe metabolic deregulation and fitness defects, resulting in a baseline slow growth rate. Helgesen et al. (2016) have shown that delta-hns strains result in slower growth kinetics, aberrant nucleoid condensation, and a structural chromosome profile that mimics a “slow-growth” state under normal conditions.

• In doing so, the authors fail to address and cite work done by other researchers on H-NS.

• This is also reflected in the “references” section, which contains too few references.

• The authors should also highlight the novelty of the work, specifically what new information was obtained compared with the known conditional growth defects of hns and the hns/stpA mutant combination.

• Please check for syntax and grammatical errors throughout the manuscript (for example, in the first paragraph of the Results section)

Experimental recommendations

1. Perform a genetic complementation assay

Δhns knockouts are highly unstable, as they can be compensated for by the bacteria acquiring secondary suppressor mutations to stay viable.

2. Perform western blotting to confirm the efficacy of the HoSeI method, proving the creation of a true null mutant and not a truncated protein.

3. Perform microscopic validation to determine whether low growth velocity correlates with structural chromosomal defects.

4. Confirm that the slow growth is not dependent on the C source. Observe the bacterial growth with alternate carbon/nitrogen sources.

**********

what does this mean?). If published, this will include your full peer review and any attached files.

If you choose “no”, your identity will remain anonymous but your review may still be made public.

**Do you want your identity to be public for this peer review?** For information about this choice, including consent withdrawal, please see our Privacy Policy

Reviewer #1: No

Reviewer #2: **Yes:**  Abhinav Saurabh

**********

[NOTE: If reviewer comments were submitted as an attachment file, they will be attached to this email and accessible via the submission site. Please log into your account, locate the manuscript record, and check for the action link "View Attachments". If this link does not appear, there are no attachment files.]

To ensure your figures meet our technical requirements, please review our figure guidelines: https://journals.plos.org/plosone/s/figures

You may also use PLOS’s free figure tool, NAAS, to help you prepare publication quality figures: https://journals.plos.org/plosone/s/figures#loc-tools-for-figure-preparation.

NAAS will assess whether your figures meet our technical requirements by comparing each figure against our figure specifications.

# Author comment: Author response to Decision Letter 1

**DOI:** 10.1371/journal.pone.0346655.r002

13 Aug 2026

Response to Reviewers'

Note on data updates: During the revision process, we conducted a rigorous re-evaluation of our growth rate calculation scripts. We identified and corrected a previous inconsistency in the calculation of instantaneous μ values. Consequently, the regression formulas (e.g., y=−0.005ln(x)+0.0026 at 37°C) and the specific growth rate data points in Fig 2 and Fig 4 have been updated. These refined values provide a more precise representation of the kinetic differences between the strains.

Response to Reviewer #1

Comment 1: Role of the Rac prophage versus the 6-kb deletion (ynaJ to abgA).

Response: We agree that the 6-kb deletion is a relevant genomic difference. However, systematic growth data from the Keio Collection—a comprehensive set of single-gene knockout mutants—confirm that none of the nine genes in this region (ynaJ, uspE, fnr, ogt, abgT, abgB, abgA, tnaB, and dcuA) individually contribute to the stimulation of growth rate. These are primarily auxiliary metabolic genes. In contrast, the Rac prophage is a recognized growth-promoting element whose presence correlates with the unique stimulation observed in MG1655.

Comment 2: Comparison of growth velocities in the W3110 background.

Response: We have expanded the Discussion to explain the comparable growth defects in WA and WA. Unlike MG1655, which utilizes a specialized strategy centered on H-NS and Rac, W3110 type A relies on a distributed network of NAPs, including HU and StpA. In this lineage, the biophysical framework is more sensitive to the loss of basic condensing agents like HU (hupB), making the structural infrastructure interdependent.

Comment 3: Need for additional experiments to support statements on page 6.

Response: To provide direct experimental support, we performed genetic complementation assays at 37°C. The specific growth rate was successfully restored in MG by an hns-expression plasmid. This confirms H-NS's direct role in growth stimulation.

Comment 4: Pleiotropic effects and evidence for H-NS as a "nucleoid structural organizer."

Response: We acknowledge the pleiotropic nature of H-NS deletions. To substantiate the structural organizer model, we conducted single-cell nucleoid imaging using DAPI staining. Our results align with the model that H-NS provides a mandatory physical scaffold for coordinating transcription and replication during the rapid onset of growth.

Minor observation 1: Instrument manufacturers and countries.

Response: We have updated the Materials and methods to include the make and country for all instruments and chemicals (e.g., Advantec [Japan], MGI Tech [China], Olympus [Japan]).

Minor observation 2: Location of Table 1.

Response: Per the journal requirements, Table 1 has been incorporated directly into the main manuscript within the Materials and methods section.

Response to Reviewer #2 (Dr. Abhinav Saurabh)

We are deeply grateful for Dr. Saurabh’s thorough review and his "Experimental recommendations," which have been instrumental in refining our model.

Comment 1: H-NS as a xenogeneic silencer.

Response: We have revised the Introduction and Discussion to explicitly acknowledge H-NS's well-established role as a global silencer of foreign/AT-rich DNA.

Comment 2: Metabolic deregulation and citation of Helgesen et al. (2016).

Response: We have now integrated Helgesen et al. (2016) as a core reference. We discuss our results in the context of their finding that hns mutants adopt a "slow-growth" type of chromosome organization even in rich media.

Comment 3: Failure to cite other researchers.

Response: We have broadened our literature review and included additional citations concerning H-NS's structural roles and its interactions with other NAPs.

Comment 4: References section size.

Response: The references section has been expanded from 14 to 18 core references, including seminal works on H-NS architecture and growth kinetics.

Comment 5: Highlighting the novelty.

Response: We have clarified the novelty of our work in the Abstract and Discussion: defining H-NS as a temperature-dependent structural prerequisite that specifically coordinates the growth-promoting potential of prophagic elements like Rac.

Comment 6: Syntax and grammatical errors.

Response: The entire manuscript, including the first paragraph of the Results, has undergone professional English language editing.

Experimental Recommendation 1: Genetic complementation assay.

Response: We have added complementation data in Fig 4a. Growth stimulation in MGΔhns was restored by the hns-expression plasmid, whereas the lack of recovery in WAΔhns reinforces our distributed network model.

Experimental Recommendation 2: Western blotting to confirm true null mutant.

Response: We performed Western blotting using anti-H-NS rabbit serum. As shown in S5 Fig, H-NS protein is completely absent in our Δhns mutants, confirming the efficacy of the HoSeI method.

Experimental Recommendation 3: Microscopic validation of chromosomal defects.

Response: We performed DAPI staining and ImageJ analysis to quantify the nucleoid-to-cell area ratio (S8 Fig). Our findings in minimal medium show that the growth defect occurs even before macroscopic condensation defects become indistinguishable, supporting the need for a fine-scale structural scaffold.

Experimental Recommendation 4: Alternate carbon/nitrogen sources.

Response: We monitored growth in M9-Glycerol (S6 Fig) and M9-Casamino acids (S7 Fig). These results demonstrate that the H-NS-mediated stimulation is environment-specific and most critical under the stringent metabolic conditions of M9-Glc.

Attachment Submitted filename: 260805 Response to Reviewers.docx

# Peer review: Decision Letter 1

**DOI:** 10.1371/journal.pone.0346655.r003

**Authors:** Saswat Mohapatra

20 Sep 2026

Structural Basis of H-NS-Mediated Temperature-Dependent Stimulation of Initial Growth in Escherichia coli.

PONE-D-26-14163R1

Dear Dr. YAMAMOTO,

We’re pleased to inform you that your manuscript has been judged scientifically suitable for publication and will be formally accepted for publication once it meets all outstanding technical requirements.

Within one week, you’ll receive an e-mail detailing the required amendments. When these have been addressed, you’ll receive a formal acceptance letter and your manuscript will be scheduled for publication.

An invoice will be generated when your article is formally accepted. Please note, if your institution has a publishing partnership with PLOS and your article meets the relevant criteria, all or part of your publication costs will be covered. Please make sure your user information is up-to-date by logging into Editorial Manager at Editorial Manager® and clicking the ‘Update My Information' link at the top of the page. For questions related to billing, please contact billing support.

If your institution or institutions have a press office, please notify them about your upcoming paper to help maximize its impact. If they’ll be preparing press materials, please inform our press team as soon as possible -- no later than 48 hours after receiving the formal acceptance. Your manuscript will remain under strict press embargo until 2 pm Eastern Time on the date of publication. For more information, please contact onepress@plos.org.

Kind regards,

Saswat Mohapatra, Ph.D.

Academic Editor

PLOS One

Additional Editor Comments (optional):

Reviewers' comments:

Reviewer's Responses to Questions

**Comments to the Author**

Reviewer #1: All comments have been addressed

Reviewer #2: All comments have been addressed

**********

2. Is the manuscript technically sound, and do the data support the conclusions?

Reviewer #1: Yes

Reviewer #2: Yes

**********

3. Has the statistical analysis been performed appropriately and rigorously?

Reviewer #1: Yes

Reviewer #2: Yes

**********

4. Have the authors made all data underlying the findings in their manuscript fully available?

The PLOS Data policy

Reviewer #1: Yes

Reviewer #2: Yes

**********

5. Is the manuscript presented in an intelligible fashion and written in standard English?

Reviewer #1: Yes

Reviewer #2: Yes

**********

Reviewer #1: (No Response)

Reviewer #2: I would like to commend the authors for their earnest effort and constructive approach in replying to my feedback. All of my comments have been answered, in addition to performing the suggested experiments.

**********

what does this mean?). If published, this will include your full peer review and any attached files.

If you choose “no”, your identity will remain anonymous but your review may still be made public.

**Do you want your identity to be public for this peer review?** For information about this choice, including consent withdrawal, please see our Privacy Policy

Reviewer #1: No

Reviewer #2: **Yes:**  Abhinav Saurabh

**********

# Editor report: Acceptance letter

**DOI:** 10.1371/journal.pone.0346655.r004

**Authors:** Saswat Mohapatra

PONE-D-26-14163R1

PLOS One

Dear Dr. YAMAMOTO,

I'm pleased to inform you that your manuscript has been deemed suitable for publication in PLOS One. Congratulations! Your manuscript is now being handed over to our production team.

At this stage, our production department will prepare your paper for publication. This includes ensuring the following:

* All references, tables, and figures are properly cited

* All relevant supporting information is included in the manuscript submission,

* There are no issues that prevent the paper from being properly typeset

You will receive further instructions from the production team, including instructions on how to review your proof when it is ready. Please keep in mind that we are working through a large volume of accepted articles, so please give us a few days to review your paper and let you know the next and final steps.

Lastly, if your institution or institutions have a press office, please let them know about your upcoming paper now to help maximize its impact. If they'll be preparing press materials, please inform our press team within the next 48 hours. Your manuscript will remain under strict press embargo until 2 pm Eastern Time on the date of publication. For more information, please contact onepress@plos.org.

You will receive an invoice from PLOS for your publication fee after your manuscript has reached the completed accept phase. If you receive an email requesting payment before acceptance or for any other service, this may be a phishing scheme. Learn how to identify phishing emails and protect your accounts at https://explore.plos.org/phishing.

If we can help with anything else, please email us at customercare@plos.org.

Thank you for submitting your work to PLOS One and supporting open access.

Kind regards,

PLOS One Editorial Office Staff

on behalf of

Dr. Saswat Mohapatra

Academic Editor

PLOS One
